# Publisher Correction: Atomically engineering activation sites onto metallic 1T-MoS_2_ catalysts for enhanced electrochemical hydrogen evolution

**DOI:** 10.1038/s41467-020-16709-4

**Published:** 2020-06-03

**Authors:** Yichao Huang, Yuanhui Sun, Xueli Zheng, Toshihiro Aoki, Brian Pattengale, Jier Huang, Xin He, Wei Bian, Sabrina Younan, Nicholas Williams, Jun Hu, Jingxuan Ge, Ning Pu, Xingxu Yan, Xiaoqing Pan, Lijun Zhang, Yongge Wei, Jing Gu

**Affiliations:** 1 Key Lab of Organic Optoelectronics & Molecular Engineering of Ministry of Education, Department of Chemistry, Tsinghua University, Beijing, 100084 P. R. China; 2 Department of Chemistry and Biochemistry, San Diego State University, 5500 Campanile Drive, San Diego, CA 92182-1030 USA; 3 State Key Laboratory of Superhard Materials, Key Laboratory of Automobile Materials of MOE, and School of Materials Science and Engineering, Jilin University, Changchun, 130012 P. R. China; 4 Department of Materials Science and Engineering, Stanford University, Stanford, CA 94305 USA; 5 UC Irvine Materials Research Institute (IMRI), University of California - Irvine, Irvine, CA 92697 USA; 6 Department of Chemistry, Marquette University, Milwaukee, WI 53201-1881 USA; 7 Collaborative Innovation Center of Advanced Nuclear Energy Technology, Institute of Nuclear and New Energy Technology, Tsinghua University, Beijing, 100084 P.R. China; 8 Department of Materials Science and Engineering, University of California - Irvine, Irvine, CA 92697 USA; 9 Department of Physics and Astronomy, University of California - Irvine, Irvine, CA 92697 USA

**Keywords:** Catalyst synthesis, Hydrogen fuel, Electrocatalysis, Two-dimensional materials

Correction to: *Nature Communications* 10.1038/s41467-019-08877-9, published online 28 February 2019.

The original version of this Article did not acknowledge Lijun Zhang as a corresponding author. This has now been corrected in the HTML version of the Article. The PDF version of the Article was correct at the time of publication.

