# Supplementary material for: Atomically engineering activation sites onto metallic 1T-MoS2 catalysts for enhanced electrochemical hydrogen evolution
Source: Nat Commun. 2019 Feb 28;10:982. doi: 10.1038/s41467-019-08877-9 (PMC6395606; doi:10.1038/s41467-019-08877-9)
Supplement: Supplementary file 1 — Supplementary Information [file 41467_2019_8877_MOESM1_ESM.pdf]

Atomically Engineering Activation Sites onto Metallic 1T-MoS_2 Catalysts for Enhanced Electrochemical Hydrogen Evolution

Supplementary Information

Huang et al.

Supplementary Figures

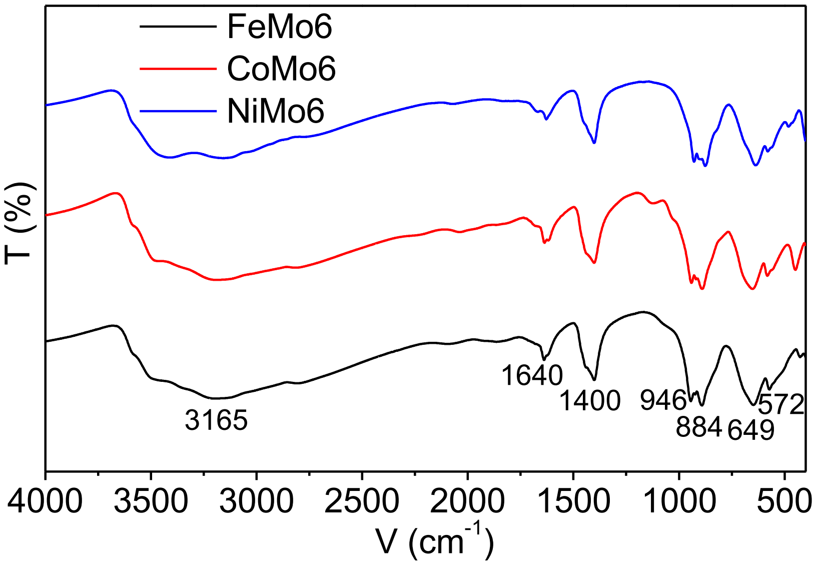

Supplementary Fig. 1 IR spectra of XMo_6 precursors. The XMo_6 precursors' peaks are quite similiar due to having similiar structures. Take $FeMo_6$ for example, the broad peaks located at ~ 3165 cm^{-1} can be ascribed to the stretching vibration of the OH and NH groups. The peaks at 1640 cm^{-1} and 1403 cm^{-1} can be attributed to the vibration of δOH and δOH, respectively. The peaks at 946 cm^{-1} and 884 cm^{-1} are associated with the Mo=O stretching vibrations. The peaks at 649 cm^{-1} and 572 cm^{-1} are attributed to asymmetric Mo-O-Mo and Fe-O-Mo stretching vibrations, respectively[1,2].

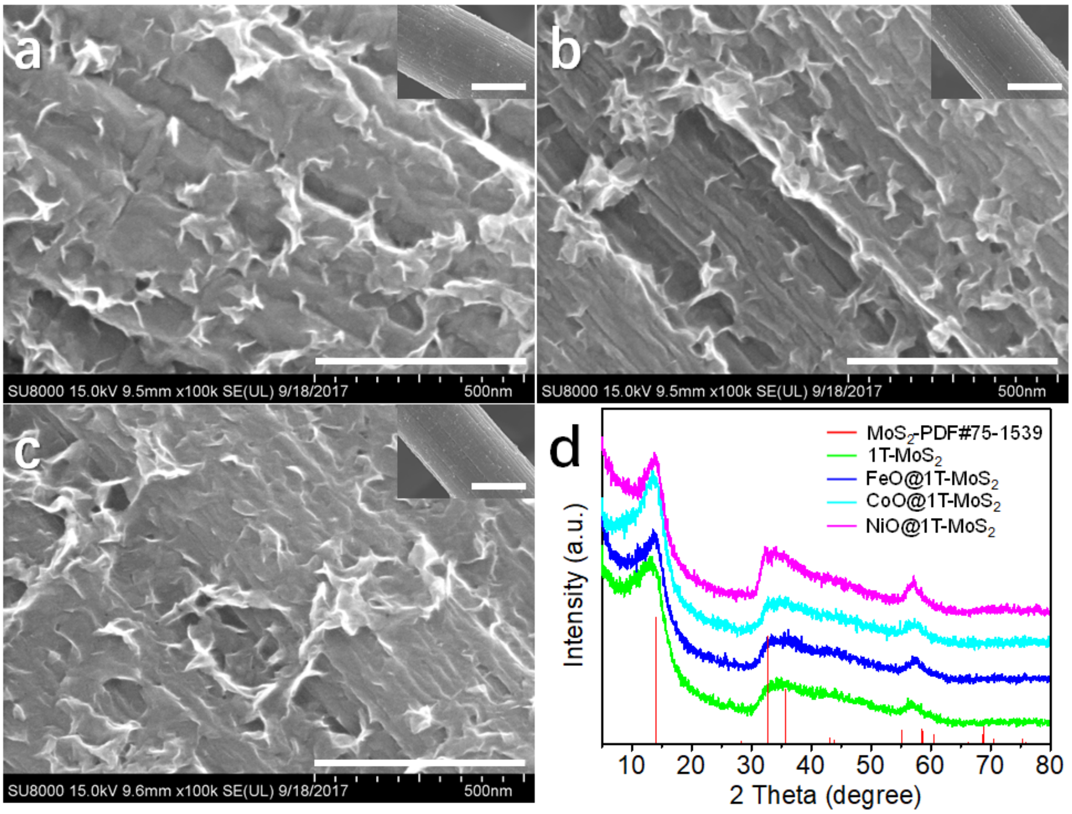

Supplementary Fig. 2 SEM images and XRD analysis of XO@1T-MoS_2. SEM images of **a**. 1T-MoS_2/CFP, **b**. FeO@1T-MoS_2/CFP, and **c**. CoO@1T-MoS_2/CFP. Scale bar, 500 nm; Scale bar of inset, 5 µm. **d**. The XRD pattern of the XO@1T-MoS_2 nanosheets. The diffraction peaks indexed to (002), (100), (102) and (110) facets of MoS_2 appear at $2\theta = 13.4°, 32.4°, 35.1°$ and $57.2°$, respectively.

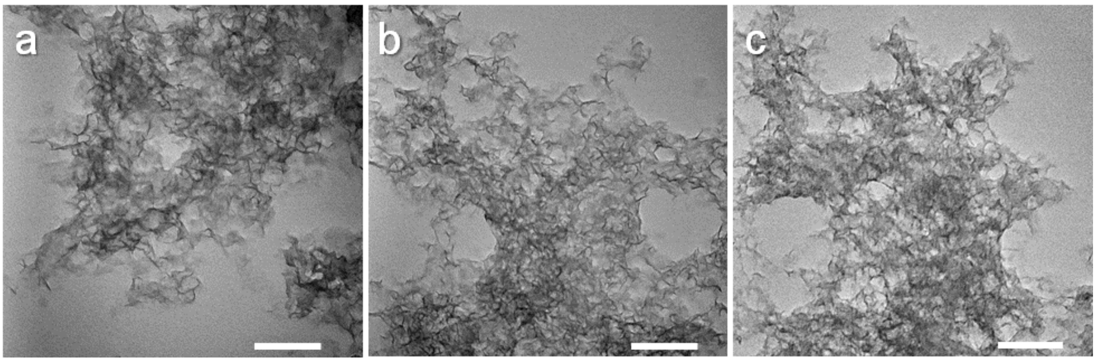

Supplementary Fig. 3 TEM images of XO@1T-MoS_2 nanosheets. **a**. 1T-MoS_2. **b**. FeO@1T-MoS_2. **c**. CoO@1T-MoS_2 nanosheets. Scale bar, 100 nm.

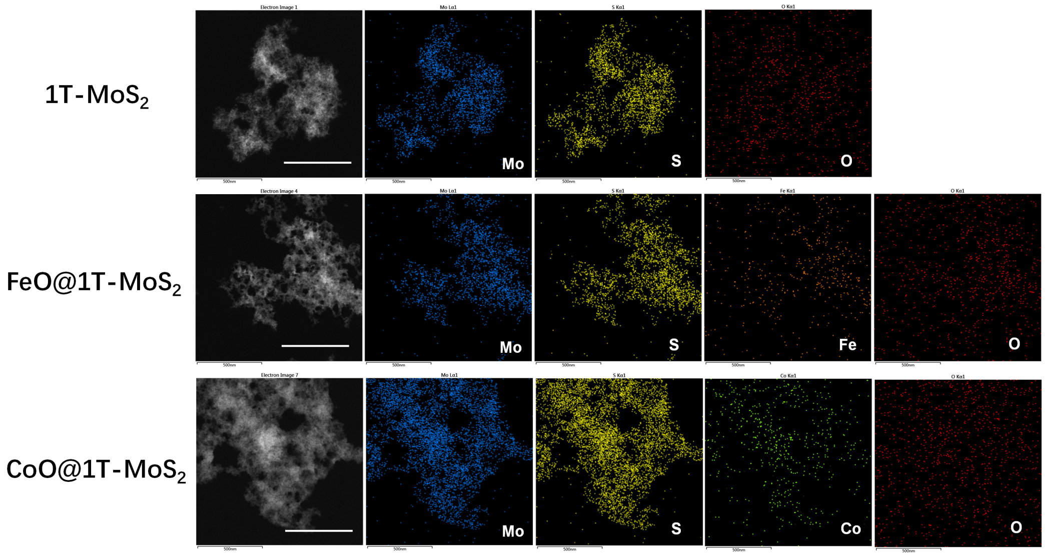

Supplementary Fig. 4 The EDS elemental mappings of XO@1T-MoS_2 nanosheets. Colour codes: Mo (blue), S (yellow), Fe (orange), Co (green) and O (red). Scale bar, 500 nm.

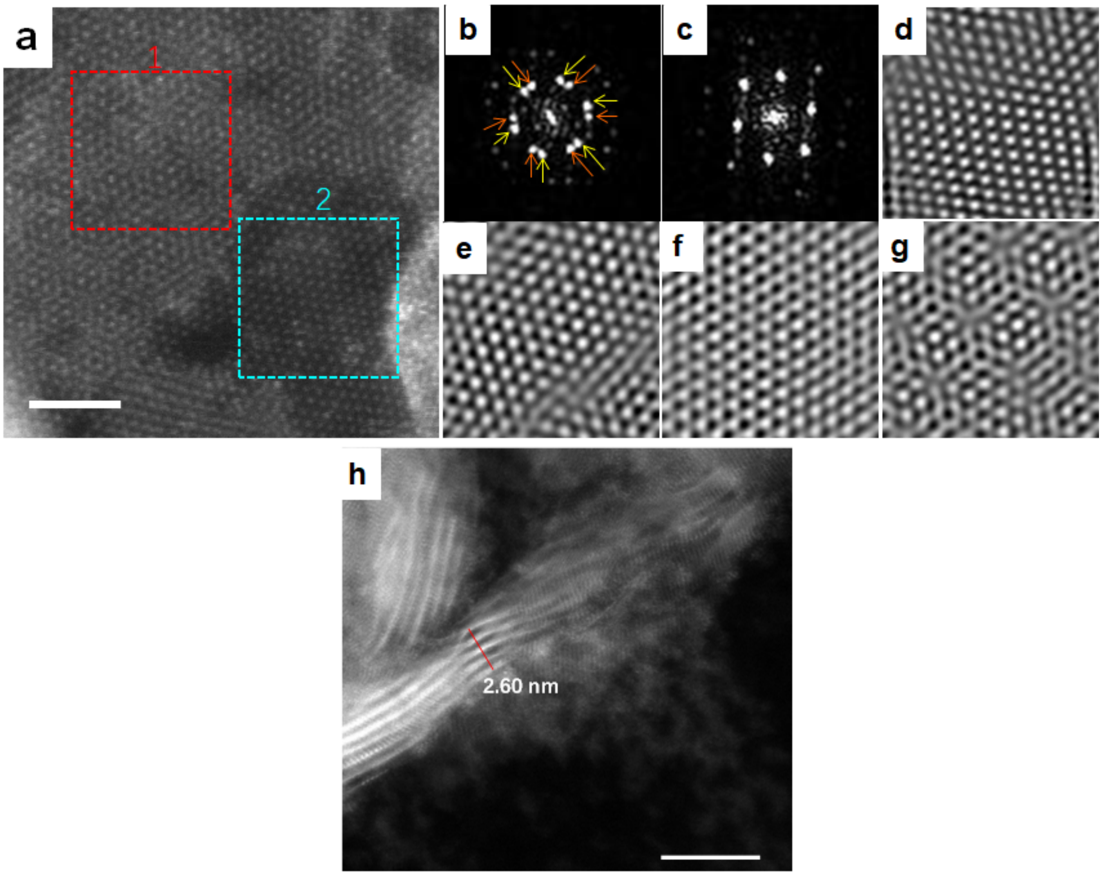

Supplementary Fig. 5 The layered structure of NiO@1T-MoS_2 nanosheets. **a**. STEM image NiO@1T-MoS_2 nanosheet (Scale bar, 2nm). **b**. Diffractogram of dotted box 1 in a; **c**. Diffractogram of dotted box 2 in a. **d**. Filtered inverse Fourier transform (IFT) of dotted box 2. **e**. IFT image of dotted box 1 using orange arrowed spots. **f**. IFT image of dotted box 1 using yellow arrowed spots. **g**. Superimposed images of e and f. **h**. The layered structure in HRTEM image of the NiO@1T-MoS_2 nanosheets (Scale bar, 5 nm). The lattice fringe spacing of NiO@1T-MoS_2 nanosheet was measured to be 2.66 nm for a five-layer nanosheet, indicating a Mo-Mo spacing of 6.50 Å.

As shown in Supplementary Fig. 5a, the top bright portion (red dotted area 1) is not a single layer region. The diffractogram analysis of the area (Supplementary Fig. 5b) shows two split set of spots, indicating the existence of two stacked twisted layers, with a 14° rotation between them. The inverse Fourier transformation images of two sets of spots (orange and yellow, Supplementary Fig. 5e-5f) demonstrate that they are both 1T-MoS_2, rather than 2H-MoS_2. Because of the twist angle, one observes the Moiré-type fringes (Supplementary Fig. 5g).

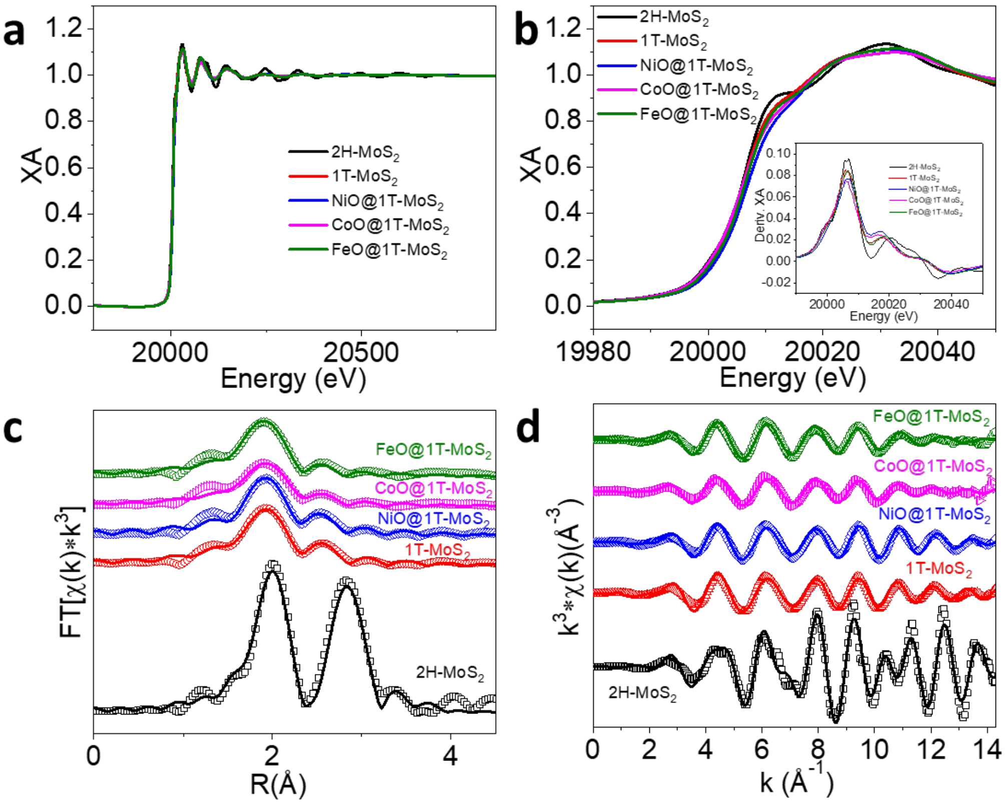

Supplementary Fig. 6 Mo K-edge XAS data and fitting. **a.** The normalized Mo K-edge EXAFS. **b.** XANES spectra of 2H-MoS_2, 1T-MoS_2, and XO@1T-MoS_2. The first derivative inset of **b** compares the XANES edge position, where it is observed that Ni has the most pronounced shift. **c**. EXAFS fitting results in R-space and **d.** K-space. For clarity in comparison, the 2H-MoS_2 and NiO@1T-MoS_2 data from Figure 3, main text, is replotted here. The corresponding fit data is presented in Supplementary Table 2.

The structural parameters around Mo atoms of XO@1T-MoS_2 were obtained by the least-squares curve parameter method with the ARTEMIS module of both IFEFFIT and USTCXAFS software packages[3]. The obtained parameters are summarized in Supplementary Table 2. The fitted R values are all within the uncertainty of the published crystallographic values. Published crystallographic distances: 2H-MoS_2 (Mo-S = 2.413; Mo-Mo = 3.150)[4]; 1T-MoS_2 (Mo-S = 2.432; Mo-Mo = 3.250)[5]. While we couldn't observe Mo-Mo in 1T-MoS_2 data, it is expected to be longer than that of 2H-MoS_2.

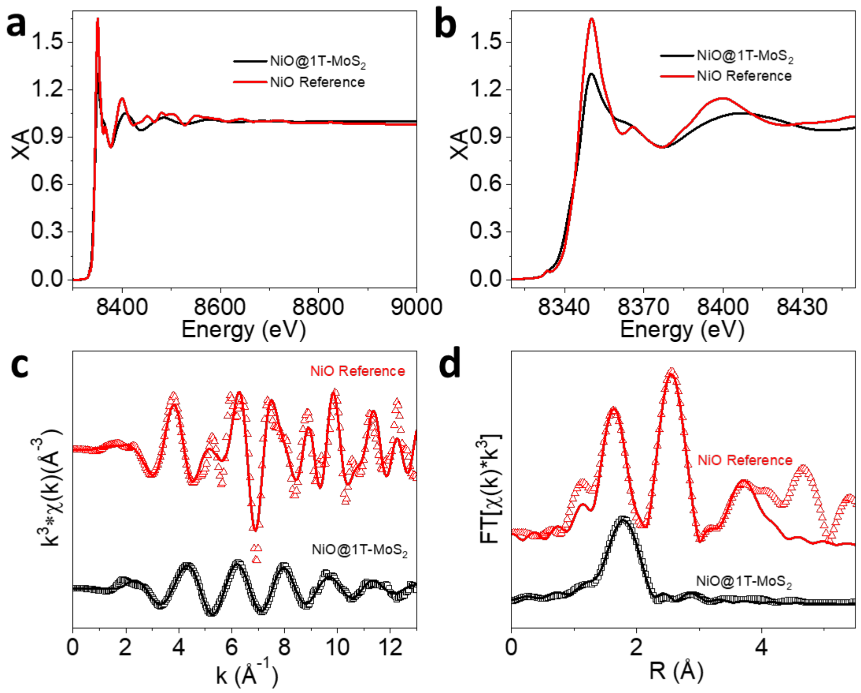

Supplementary Fig. 7 Ni K-edge of NiO@1T-MoS_2 and NiO reference comparison. **a.** Comparison of the Ni K-edge XAS spectra in the EXAFS and **b.** XANES energy ranges. **c.** The K-space and **d.** R-space comparisons with data shown in open points and best fits in solid lines. The corresponding NiO reference fit parameters are shown in Supplementary Table 3.

The structural parameters around Ni atoms of NiO@1T-MoS_2 as a function of synthesis temperature were fit using the same model utilized at Mo K-edge, by replacing Mo with Ni. The FEFF fitting of the NiO reference spectrum was performed to R=4, requiring second and third-shell single and multiple-scattering paths. The first-shell Ni-O and second-shell Ni-Ni distances both fit to values within the uncertainty of the published crystal structure that was used for the model.[6]

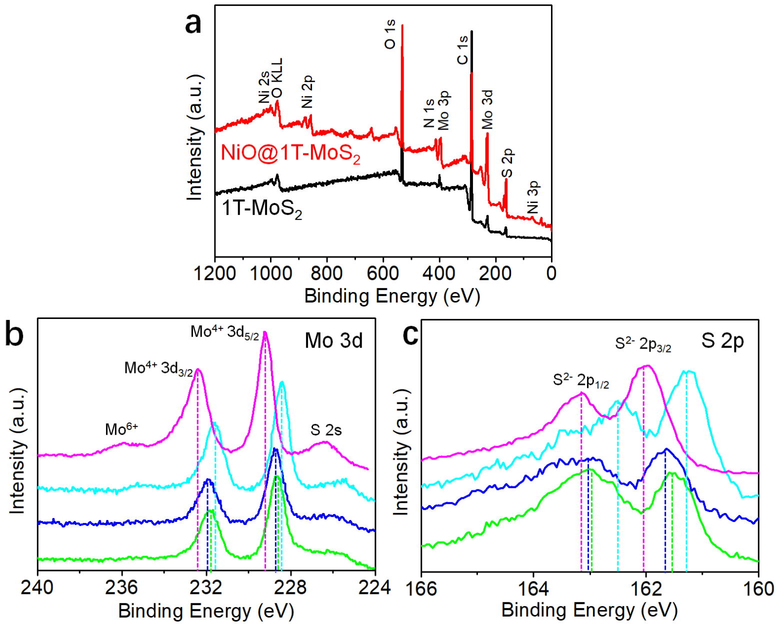

Supplementary Fig. 8 XPS spectra of the as-prepared XO@1T-MoS_2 and 1T-MoS_2 nanosheets. **a**. Wide-scan survey XPS spectra of NiO@1T-MoS_2 and 1T-MoS_2 nanosheets; **b**. High-resolution XPS signal of Mo 3d in XO@1T-MoS_2 (colour code: green: 1T-MoS_2; blue: FeO@1T-MoS_2; light blue: CoO@1T-MoS_2; purple: NiO@1T-MoS_2). **c**. High-resolution XPS signal of S 2p in XO@1T-MoS_2 (colour code: green: 1T-MoS_2; blue: FeO@1T-MoS_2; light blue: CoO@1T-MoS_2; purple: NiO@1T-MoS_2).

For the bare 1T-MoS_2 nanosheets, the peaks of Mo $3d_{5/2}$ and Mo $3d_{3/2}$ appear at 228.63 and 231.77 eV and the peaks of S $2p_{3/2}$ and S $2p_{1/2}$ are located at 161.51 and 162.98 eV, respectively. For FeO@1T-MoS_2 nanosheets, the peaks of Mo $3d_{5/2}$ and Mo $3d_{3/2}$ upshift slightly to 228.71 and 231.90 eV, respectively, and the peaks of S $2p_{3/2}$ and S $2p_{1/2}$ also upshift slightly to 161.64 and 163.08 eV, respectively. For CoO@1T-MoS_2 nanosheets, the peaks of Mo $3d_{5/2}$ and Mo $3d_{3/2}$ downshift to 228.42 and 231.63 eV, respectively. Similarly, the peaks of S $2p_{3/2}$ and S $2p_{1/2}$ downshift to 161.23 and 162.44 eV, respectively. For NiO@1T-MoS_2 nanosheets, the peaks of Mo $3d_{5/2}$ and Mo $3d_{3/2}$ upshift to 229.22 and 232.41 eV, respectively and the peaks of S $2p_{3/2}$ and S $2p_{1/2}$ also upshift to 162.02 and 163.23 eV, respectively. Due to the large amount present of S^{2-} and the corresponding Fe, Co and Ni XPS, Fe, Co, and Ni are mainly existing in the oxidation state of 2+. The dopant mechanism might be different in between Fe/Ni and Co. The upshift of the peaks is usually attributed to the n-doping process, which causes a Fermi level shift towards the conduction band edge or electron transfer from 1T-MoS_2 to dopant[7]. While the downshift is indicative of p-doping process, which indicates reduction of 1T-MoS_2 due to the transfer of electron from the dopants[8].

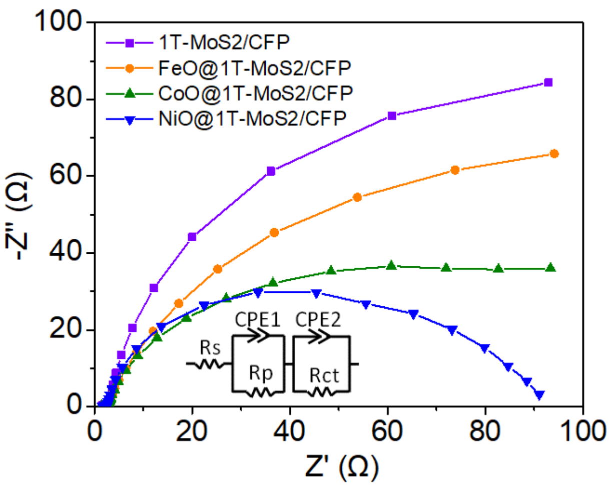

Supplementary Fig. 9 Electrochemical impedance spectroscopy for XO@1T-MoS_2/CFP. Electrochemical impedance spectroscopy for 1T-MoS_2/CFP and XO@1T-MoS_2/CFP are obtained at an overpotential of 200 mV in 1.0 M KOH. The simplified equivalent circuits are applied to fit the ESI in the inset, where the Rs, Rp, Rct represent the electrolyte, electrode porosity, charge transfer resistance, respectively. CPE is the constant phase angle element, which represents the double layer capacitance of solid electrode in a real-world situation.

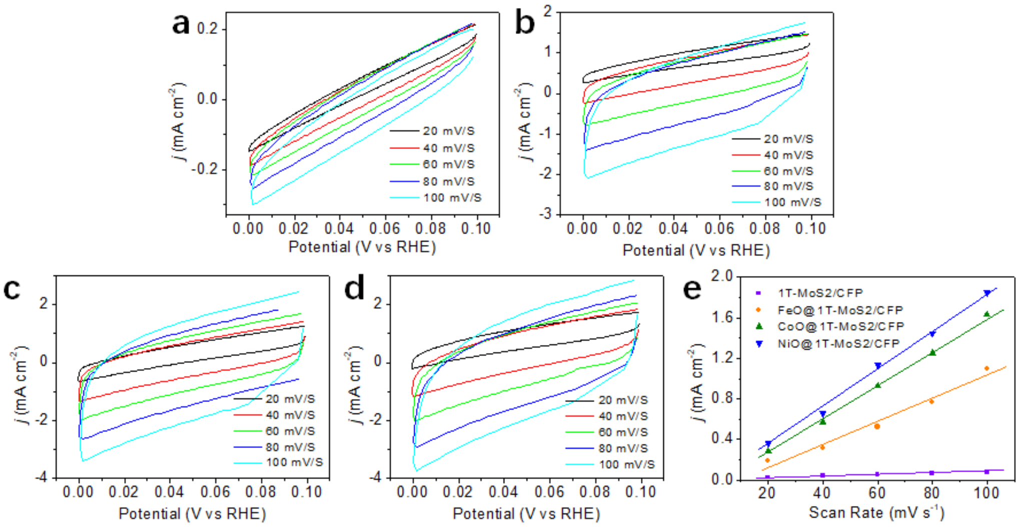

Supplementary Fig. 10 Cyclic voltammograms and C_{dl} estimation of 1T-MoS_2/CFP and XO@1T-MoS_2/CFP electrocatalysts. Cyclic voltammograms of **a**. 1T-MoS_2/CFP, **b**. FeO@1T-MoS_2/CFP, **c**. CoO@1T-MoS_2/CFP, and **d**. NiO@1T-MoS_2/CFP with different scan rates from 20 to 100 mV s^{-1} in the potential range of 0-100 mV (*vs* RHE) in 1.0 M KOH. **e**. Estimation of C_{dl} by plotting the current density variation ($\Delta j = (j_a–j_c) / 2$, at 50 mV vs. RHE) against scan rate to fit the linear regression.

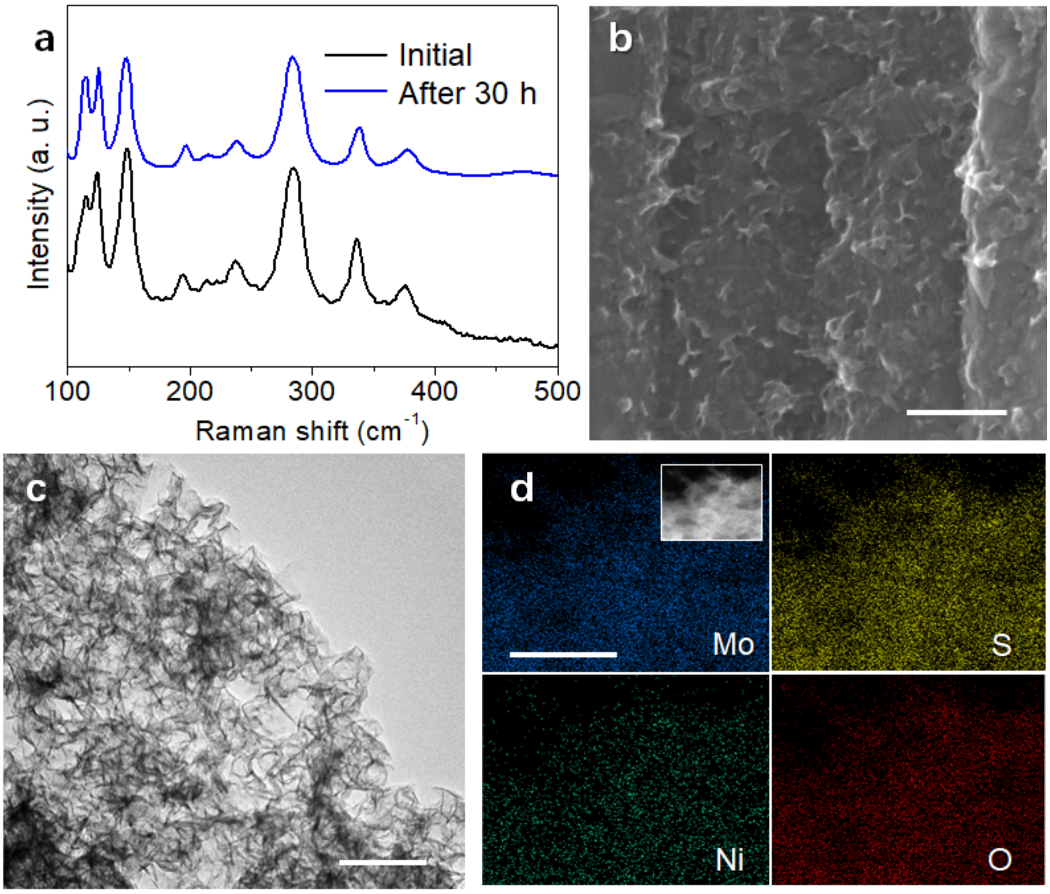

Supplementary Fig. 11 Characterization of NiO@1T-MoS_2/CFP after long-term HER stability testing. **a**. The Raman patterns of NiO@1T-MoS_2/CFP initial (black curve) and after 30 hours (blue curve) in 1.0 M KOH for HER. **b**. SEM image (Scale bar, 200 nm). **c**. TEM image (Scale bar, 100 nm), and **d**. EDS mappings of Mo, Ni, S, O eleements in NiO@1T-MoS_2/CFP after long-term HER stability testing in 1.0 M KOH (Scale bar, 250 nm).

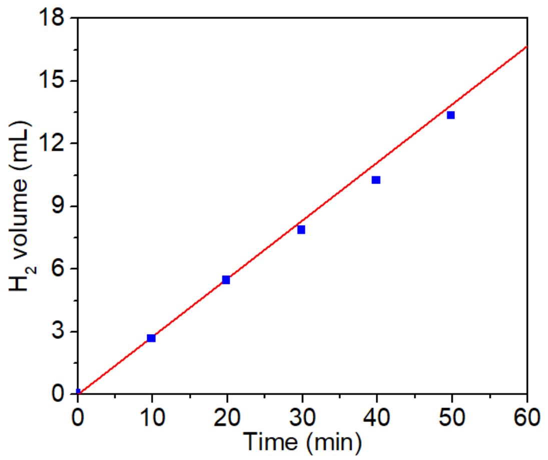

Supplementary Fig. 12 Faradaic efficiency testing for NiO@1T-MoS_2/CFP. The amount of H_2 theoretically calculated and experimentally measured versus time of HER on the NiO@1T-MoS_2/CFP electrocatalyst with a current of 60 mA.

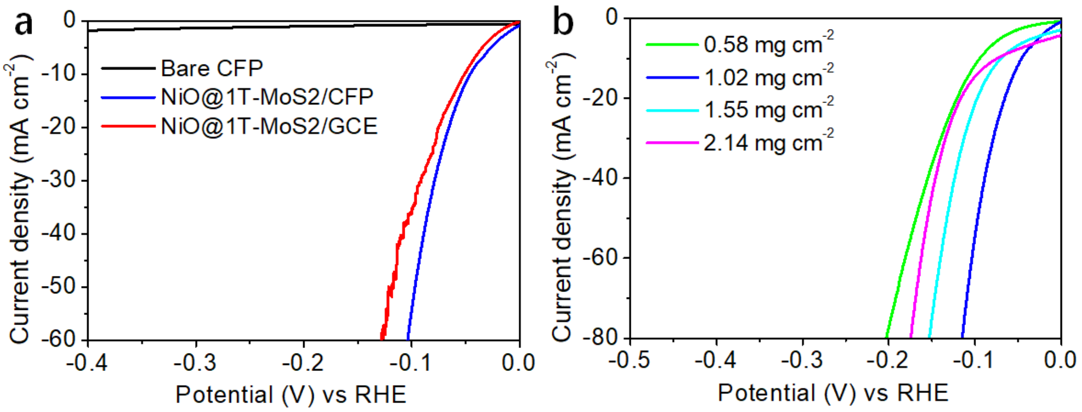

Supplementary Fig. 13 The influence of substrates and mass loading. The influence of substrates and mass loading. **a**. Polarization curves of the bare CFP (black), NiO@1T-MoS_2/CFP (blue) and NiO@1T-MoS_2/GCE (red). The catalysts' mass of 1 mg cm^{-2} were loaded by dispersing suitable amounts of slurry from 1.0 mL of solution containing 10 mg of NiO@1T-MoS_2, 0.95 mL of ethanol and 50 uL of 0.5 wt% Nafion. The HER performance was measured in 1.0 M KOH aqueous solution at a scan rate of 5 mV s^{-1}. **b**. Polarization curves of NiO@1T-MoS_2/CFP under different mass loadings. The mass loadings, obtained by inductively coupled plasma atomic emission spectroscopy (ICP-AES), on CFP can be changed by adjusting the concentration of NiMo6 precursor and TAA. The mass loading of 0.13, 0.58, 1.02, 1.55 and 2.14 mg·cm^{-2} was obtained by reacting 5 , 30, 50, 60 and 80 mg NiMo6 with 8, 48, 80, 96, 128 mg TAA in 10 mL aqueous solution at 180 °C for 24 hours, respectively.

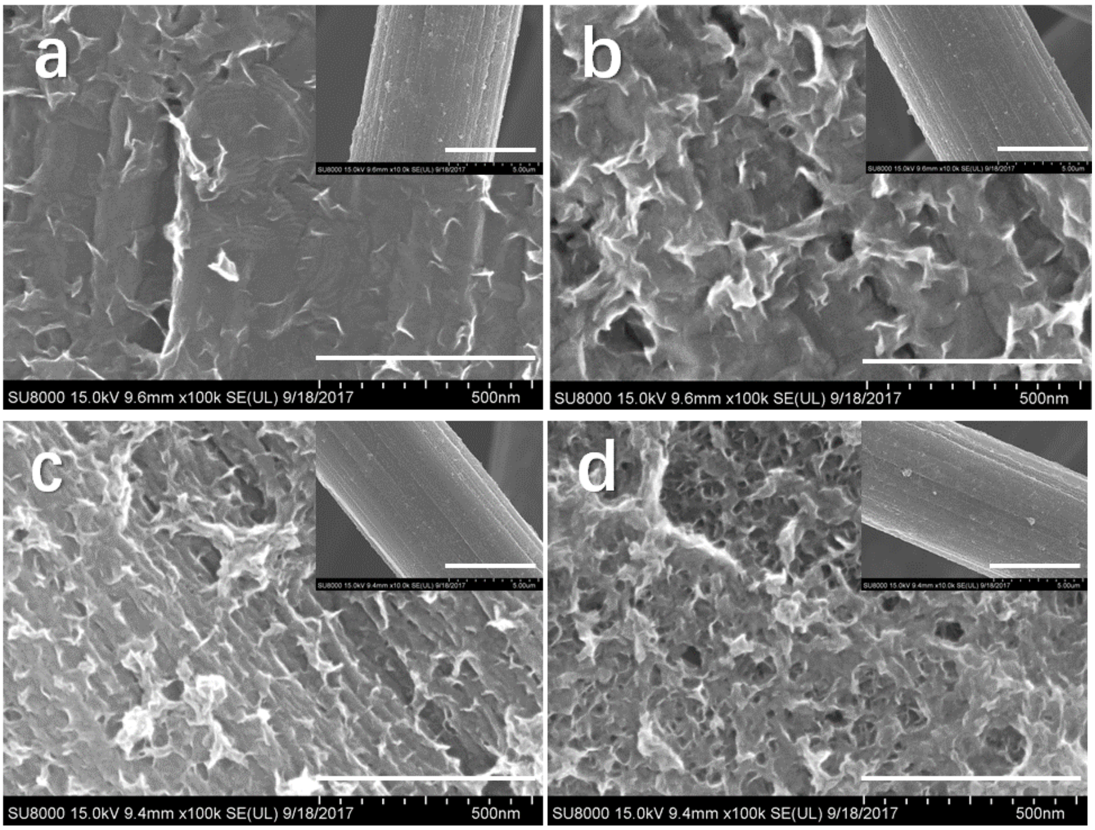

Supplementary Fig. 14 The SEM images of NiO@1T-MoS_2/CFP electrocatalysts with different mass loadings. **a**. 0.58 mg cm^{-2}. **b**. 1.02 mg cm^{-2}. **c**. 1.55 mg cm^{-2}. **d**. 2.14 mg cm^{-2}. Scale bar, 500nm; Scale bar of inset, 5 μm.

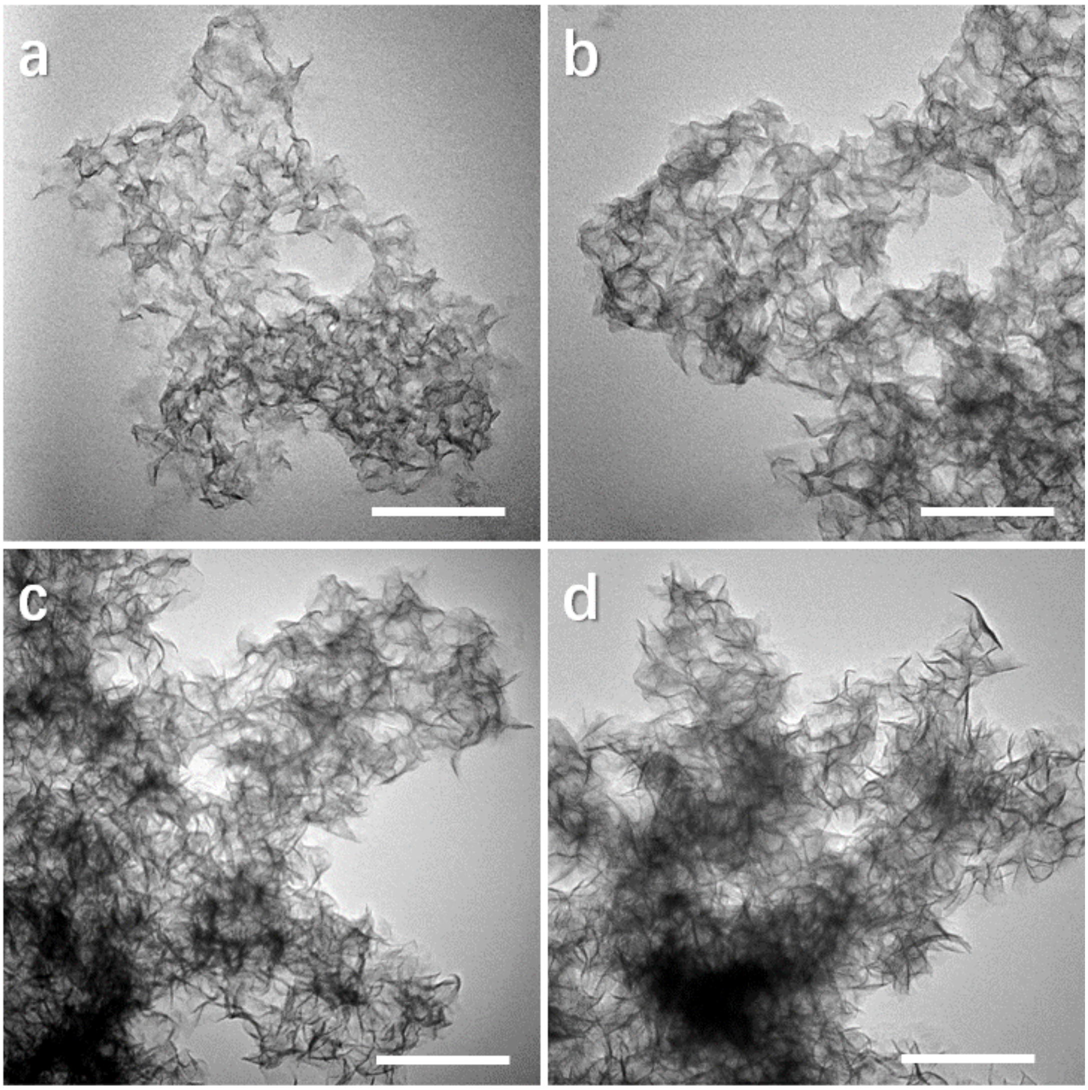

Supplementary Fig. 15 The TEM images of NiO@1T-MoS_2 nanosheets synthesized at different temperatures. **a**. T-160. **b**. T-180. **c**. T-200. **d**. T-220. Scale bar, 100 nm.

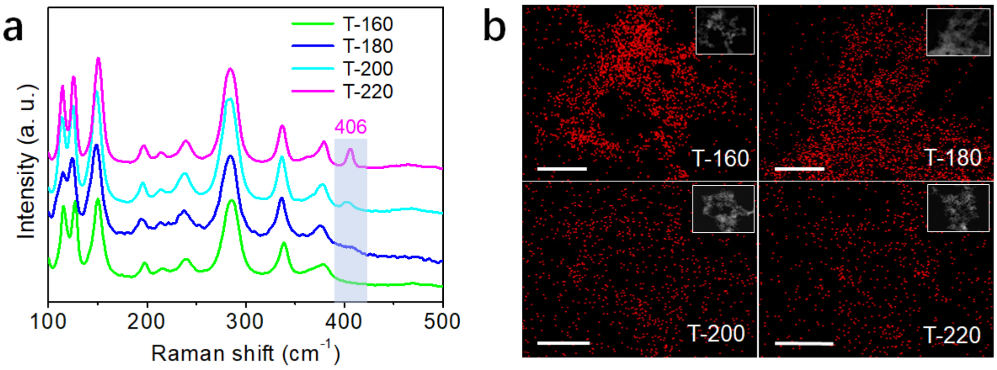

Supplementary Fig. 16 The Raman spectra and EDS results of T-n nanosheets. **a**. The Raman spectra of NiO@1T-MoS_2 nanosheets (denoted as T-n, where n denotes the synthesis temperature in °C) synthesized at different temperatures. **b**. EDS results showing oxygen contents of different T-n samples. Scale bar, 100 nm. The accurate oxygen contents are summarized in Supplementary Table 1.

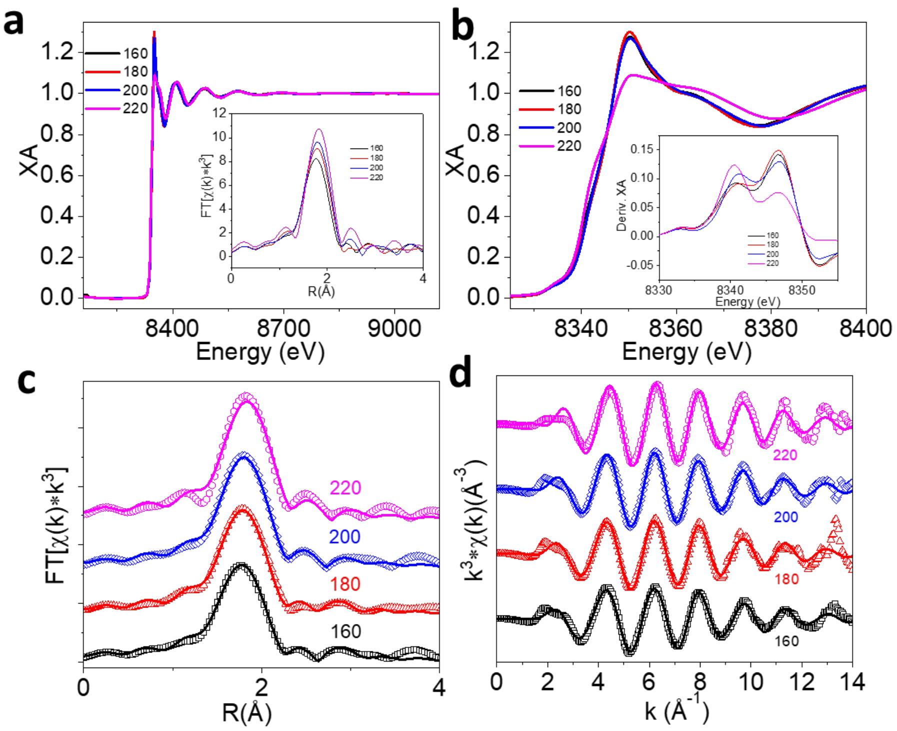

Supplementary Fig. 17 Synthesis temperature-dependent XAS at Ni K-edge. **a.** Ni K-edge EXAFS and **b.** XANES spectra for NiO@1T-MoS_2 synthesized at different temperatures, in Celsius degrees. Inset of **a** compares R-space spectra, inset of **b** compares first derivative spectra. **c.** The best fits in R-space and **d.** K-space with the corresponding data shown in Supplementary Table 5. For the sake of comparison, the NiO@1T-MoS_2 data in Fig.3 from main text, is replotted here (synthesis temperature of 180 Celsius degree).

The structural parameters around Ni atoms of NiO@1T-MoS_2 as a function of synthesis temperature were fit using the same model utilized at Mo K-edge, by replacing Mo with Ni. Increased synthesis temperature results in an increased coordination number of S. Due to the relatively small scattering intensity of O, Ni-O paths could not be reasonably incorporated into the fitting model. Due to both the presence of O and the local structural environment of Ni, Ni-Mo scattering was observed and Ni-Mo was incorporated into the model. However, the apparent value of N is quite small, suggesting that while Ni-Mo is observed, Ni is likely saturatively coordinated in a S-rich environment, which is supported by EXAFS fit parameters. While the expected highest value of N is observed in T-160, the sample with the highest O content and least S content, there is no strong trend due to large uncertainty in N value. However, the clear observation of Ni-Mo single scattering in all samples suggests the presence of Ni-O-Mo moieties.

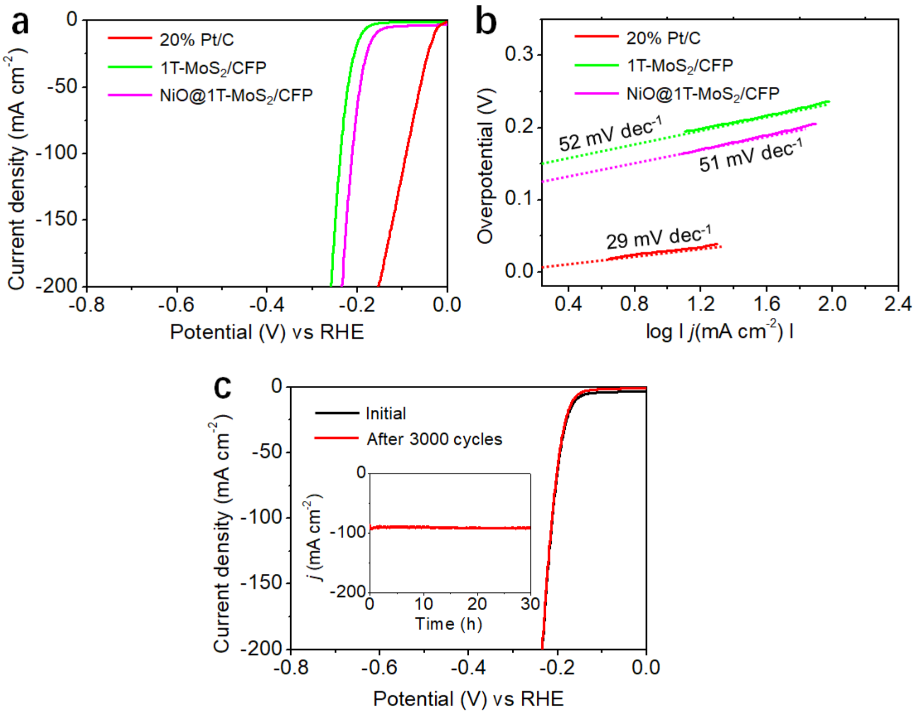

Supplementary Fig. 18 HER performance of NiO@1T-MoS_2/CFP in acidic conditions. **a.** Polarization curve of catalysts in 0.5 M H_2SO_4 (scan rate: 5 mV s-1 under a three-electrode configuration). **b.** Corresponding Tafel plots. c. Stability testing of NiO@1T-MoS_2 in 0.5 M H_2SO_4 (inset: the long-term durability tests at η = 210 mV for 30 hours).

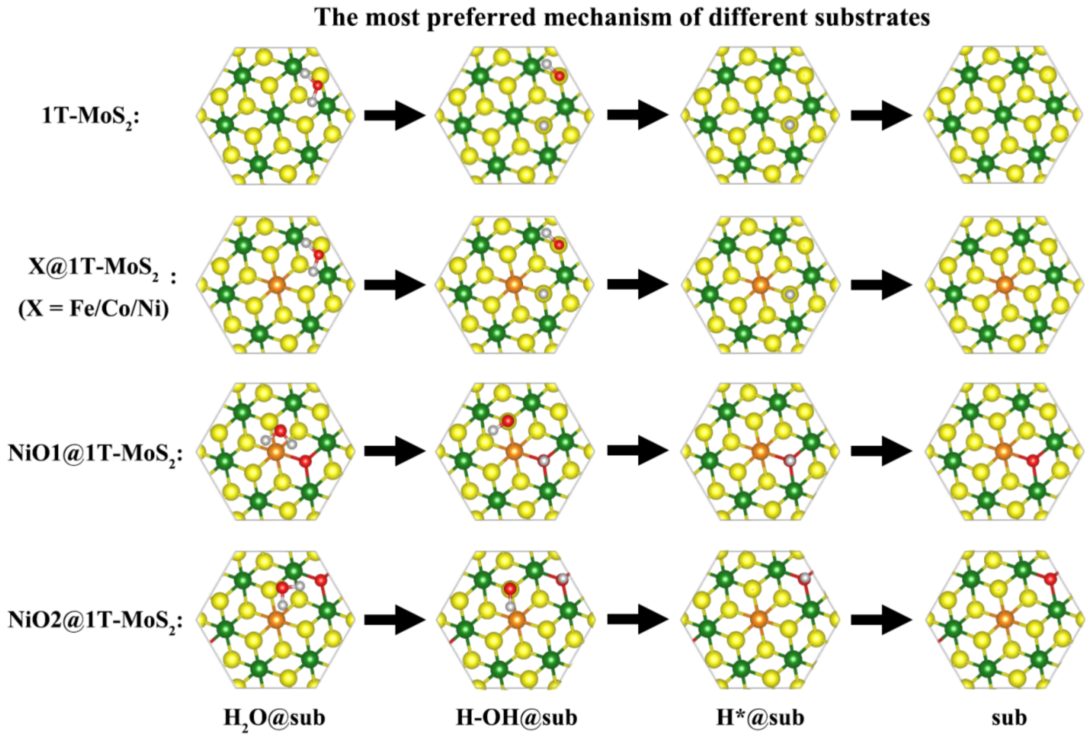

Supplementary Fig. 19 HER reaction pathways and relevant structures of the most possible intermediate steps on the surface of 1T-MoS_2, X@1T-MoS_2 (X=Fe/Co/Ni), NiO1@1T-MoS_2, and NiO2@1T-MoS_2, respectively.

To better describe the reaction pathway of the theoretical calculation, we use the hexagonal motif as a model to show the evolution of atomic adsorption on top of different substrates. For each substrate, we have tested many possible intermediate states to find out the most energetic stable one. The results shown here are the most energetic preferred mechanisms. Firstly, for pure 1T-MoS_2, H* is on top of the sulfur atom and the OH* locates at the nearest sulfur atom. Many equivalent adsorption sites for the processes of water dissociation and hydrogen generation exist. Here we list only one of them. This is also applied to the following description. Secondly, for Fe/Co/Ni doped 1T-MoS_2 (X@1T-MoS_2), H* prefers to adsorb on the sulfur atom of …X-S-Mo… bond, with the OH* located at the nearest sulfur atom of …Mo-S-Mo… bonding. Lastly, for NiO1@1T- MoS_2 and NiO2@1T-MoS_2, H* prefers to adsorb on the top of doped oxygen atom rather than the sulfur atom, while for the OH*, different from that in X@1T-MoS_2, located upon the sulfur atom of …Ni-S-Mo… bonding. As we know, the complexity of chemical reaction is hard to be described with a single pathway, so the most possible mechanism on each substrate provides the most likely reaction processes for water dissociation and hydrogen generation.

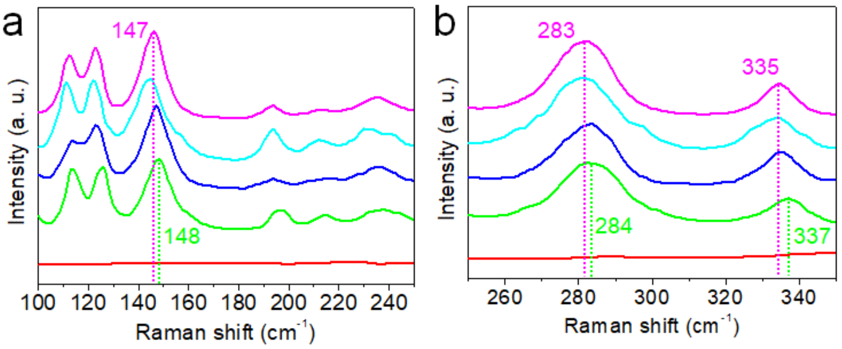

Supplementary Fig. 20 Raman spectra at different ranges. **a**. Raman spectra of the XO@1T-MoS_2 nanosheet at a range of 100-250 cm^{-1}. **b**. Raman spectra of the XO@1T-MoS_2 nanosheet at a range of 250-350 cm^{-1}. (red: 2H-MoS_2; green: 1T-MoS_2; blue: FeO@1T-MoS_2; light blue: CoO@1T-MoS_2; pink: NiO@1T-MoS_2).

We have retested and reanalyzed the Raman spectra of all samples. The spectrum did not show obvious changes at a scale of 100-500 cm^{-1}. However, as we narrow the range to 100-250 cm^{-1} and 250-350 cm^{-1}, the samples doped with secondary metals (Fe, Co, Ni) do show slight shifts in comparison with undoped 1T-MoS_2. For example, the selected phonon Raman peaks of NiO@1T-MoS_2 (pink line) is located at 147, 283, and 335 cm^{-1}, while that of 1T-MoS_2 (green line) are 148, 284, and 337 cm^{-1}. Considering these small changes, we may conclude that the introduction of secondary metals (Fe, Co, and Ni) only change the electronic properties of the 1T-MoS_2 structure slightly (Fig. 4a), consistent with XAS and XRD results.

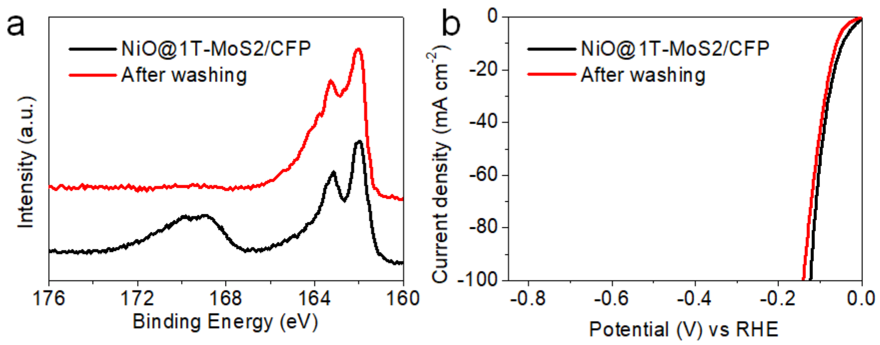

Supplementary Fig. 21 The influence of S^{6+} (sulfate moieties) on HER performance. **a**. XPS spectra of S2p for the as-prepared NiO@1T-MoS_2/CFP and that after washing. **b**. The HER performance of NiO@1T-MoS_2/CFP and that after washing. Note: The as-prepared NiO@1T-MoS_2/CFP sample is washed by 8 mL deionized water with shaking (repeat 10 times) to remove the sulfate moieties.

Our further experimental results show that the big S^{6+} peak is derived from the sulfate moieties physically absorbed on the surface of planar 2D 1T-MoS_2 sheets, which can be removed by washing with deionized water (Supplementary Fig. 21a). Furthermore, the removal of sulfate moieties has little influence on the HER performance of NiO@1T-MoS_2/CFP (Supplementary Fig. 21b).

Supplementary Tables

Supplementary Table 1 Summary of elemental analysis of varied XO@1T-MoS_2 electrocatalysts

XO@1T-MoS_2 Catalysts	Mo content (wt. %)[a]	S content (wt. %)[a]	Mo/S Atomic ratio	X=Fe content (wt. %)[a]	X=Co content (wt. %)[a]	X=Ni content (wt. %)[a]	X/Mo Atomic ratio	N content (wt. %)[b]	O content (wt. %)[c]
1T-MoS_2	50.87	35.48	1:2.09	-	-	-		6.03	3.61
FeO@1T-MoS_2	45.98	34.95	1:2.27	4.51	-	-	1:5.93	7.27	4.81
CoO@1T-MoS_2	44.29	35.37	1:2.39	-	4.58	-	1:5.94	7.55	4.52
NiO@1T-MoS_2 (T-180)	43.85	34.49	1:2.35	-	-	4.50	1:5.96	7.34	4.59
NiO@1T-MoS_2 (T-160)	41.98	34.20	1:2.44	-	-	4.41	1:5.83	8.63	6.38
NiO@1T-MoS_2 (T-200)	46.90	35.13	1:2.24	-	-	4.68	1:6.13	5.47	2.52
NiO@1T-MoS_2 (T-220)	49.76	35.61	1:2.14	-	-	4.99	1:6.10	2.41	1.35

[a] Data are determined by ICP-AES elemental analysis; [b] Data are determined from C, H and N elemental analysis; [c] Data are calculated from results of [a] and [b].

Supplementary Table 2 Summary of local structural parameters in electrocatalysts fitted from Mo K-edge EXAFS data

Sample	Path	R (Å)	N	σ^2 (Å^2)	ΔE (eV)
2H-MoS_2	Mo-S	2.41	6.00	0.004	0.99
	Mo-Mo	3.17	6.00	0.005	
	Mo-S	4.00	6.00	0.010	
1T-MoS_2	Mo-S	2.44	6.00	0.01	3.91
	Mo-O	2.86	3.52	0.001	-6.00
FeO@1T-MoS_2	Mo-S	2.41	4.43	0.008	-0.46
	Mo-O	2.86	2.00	0.005	-3.48
CoO@1T-MoS_2	Mo-S	2.42	4.50	0.01	0.235
	Mo-O	2.85	1.91	0.001	-10.00
NiO@1T-MoS_2	Mo-S	2.43	4.13	0.006	3.33
	Mo-O	2.84	2.95	0.001	-4.87

The structural parameters around the Mo atoms of XO@1T-MoS_2 were obtained by the least-squares curve parameter method with the ARTEMIS module of both IFEFFIT and USTCXAFS software packages[3]. The obtained parameters are summarized in Supplementary Table 2. The fitted R values are all within the uncertainty of the published crystallographic values. Published crystallographic distances: 2H-MoS_2 (Mo-S = 2.413; Mo-Mo = 3.150)[4]; 1T-MoS_2 (Mo-S = 2.432; Mo-Mo = 3.250)[5]. While we couldn't observe Mo-Mo in 1T-MoS_2 data, it is expected to be longer than that of 2H-MoS_2.

The additional Mo-O path included was required in order to sufficiently fit the R-space peak directly adjacent to the first-shell scattering peak. Because the position of the peak is much too short to be due to Mo-Mo single scattering, and because the amplitude of the peak is relatively small, this was assigned as a single-scattering peak to a small scattering atom, such as O. We assume that these atoms are chemisorbed on the surface of the planar 2D 1T-MoS_2 sheets between stacked layers of material. Interestingly, it was found in XPS that sulfate moieties may be present in the structure, and these may very well be the source of Mo-O scattering observed in EXAFS.

Note: R is the length of the vector, N is the coordination number, σ^2 is Debye-Waller factor and ΔE is the edge-energy shift. The obtained R values are approximately ±0.02 Å.

Supplementary Table 3 Local structure parameters for NiO reference spectrum at Ni K-edge

Vector	N	σ^2 (Å^2)	E_0	R (Å)
Ni-O	6	0.0027	-1.789	2.06
Ni-Ni	12	0.0096	-6.765	2.94
Ni-O-Ni	48	0.0078	-6.765	3.37
Ni-O	8	0.0100	-6.765	3.50
Ni-Ni	6	0.0047	-3.447	4.05
Ni-O-Ni	12	0.0051	-3.447	4.15
Ni-O-Ni-O	6	0.0100	-3.447	4.22

FEFF fitting of the NiO reference spectrum was performed to R=4, requiring second and third-shell single and multiple-scattering paths. The first-shell Ni-O and second-shell Ni-Ni distances both fit to values within uncertainty of the published crystal structure that was used for the model.[6] In comparison with the Ni-K edge fit results in Supplementary Table 3, it is observed that, first of all, the first-shell distance is significantly shorter in NiO compared to NiO@-T-MoS_2 due to S ligands in the latter. Importantly, the second-shell feature is assigned to Ni-Ni in NiO (2.94 Å) and Ni-Mo in NiO@1T-MoS_2 (3.05 Å). These two distances are significantly different and demonstrate that Ni is incorporated into the 1T-MoS_2 lattice, and does not form discrete NiO phase regions in the material.

Supplementary Table 4 Summary of the HER activity of electrocatalysts in 1 M KOH

Catalysts	η_{10} (mV)	η_{onset} (mV)	Tafel slope (mV dec^{-1})	R_p [a] (Ω)	R_{ct} [a] (Ω)	CPE1[a]		CPE2[a]		C_{dl} [b] (mF cm^{-2})	j_0 [c] (mA cm^{-2})
						CPE1-P	CPE1-T	CPE2-P	CPE2-T		
1T-MoS_2/CFP	219	133	107	89.41	210.6	0.85	0.002	0.53	0.002	1.09	1.3 x 10^{-1}
FeO@1T-MoS_2/CFP	187	105	106	55.86	149.5	089	0.002	0.61	0.003	11.38	1.4 x 10^{-1}
CoO@1T-MoS_2/CFP	117	52	84	35.57	106.1	0.95	0.002	0.78	0.003	16.37	1.8 x 10^{-1}
NiO@1T-MoS_2/CFP	46	~ 0	52	23.65	65.5	1.09	0.002	0.85	0.005	18.32	4.4 x 10^{-1}
20% Pt/C/CFP	23	~ 0	32		-					-	6.9 x 10^{-1}

[a] Data are measured at η = 200 mV. Notes: the R_s, R_p, R_{ct} and CPE represent the electrolyte, electrode porosity, charge transfer resistance, and the constant phase angle element, respectively. [b] Data are calculated according to the CV results in Supplementary Fig. 10. [c] Exchange current densities (j_0) are obtained from Tafel curves by using the extrapolation method according to the following equation: $\eta = a + b \log j$, where a is the intercept on the y-axis and b is Tafel slope. Exchange current density (j_0) is calculated when η = 0 V.

Supplementary Table 5 Summary of local structural parameters in NiO@1T-MoS_2 synthesized at different temperatures

	Ni –S Single Scatter				Ni-Mo Single Scatter			
Temp.	E_0	σ^2 (Å^2)	N	R (Å)	E_0	σ^2 (Å^2)	N	R (Å)
160	-10.2	0.008	3.99	2.20	-6.5	0.01	0.56	3.10
180	-8.5	0.008	4.27	2.21	-10	0.01	0.40	3.05
200	-7.0	0.008	4.51	2.22	-10	0.01	0.42	3.14
220	-2.0	0.008	4.90	2.23	-10	0.01	0.50	3.11

Note: The above structural parameters were fitted from Ni K-edge EXAFS data. R is the length of the vector, N is the coordination number, σ^2 is Debye-Waller factor and ΔE is the edge-energy shift. The obtained R values are approximately ±0.02 Å.

The structural parameters around Ni atoms of NiO@1T-MoS_2 as a function of synthesis temperature were fit using the same model utilized at Mo K-edge, by replacing Mo with Ni. Increased synthesis temperature results in an increased coordination number of S. Due to the relatively small scattering intensity of O, Ni-O paths could not be reasonably incorporated into the fitting model. Due to both the presence of O and the local structural environment of Ni, Ni-Mo scattering was observed and Ni-Mo was incorporated into the model. However, the apparent value of N is quite small, suggesting that while Ni-Mo is observed, Ni is likely saturatively coordinated in a S-rich environment, which is supported by EXAFS fit parameters. While the expected highest value of N is observed in T-160, the sample with the highest O content and least S content, there is no strong trend due to large uncertainty in N value. However, the clear observation of Ni-Mo single scattering in all samples suggests the presence of Ni-O-Mo moieties.

Supplementary Table 6 The key values of water dissociations and hydrogen generations on the surface of the energetic stable structures for different substrates

	**Δ*E*(H_2O)/eV**	**Δ*ZPE*(H_2O)/eV**	**Δ*TS*(H_2O)/eV**	**Δ*G*(H_2O)/eV**
**1T-MoS_2**	1.211	-0.008	-0.447	1.650
**Fe@1T-MoS_2**	0.579	-0.013	-0.439	1.005
**Co@1T-MoS_2**	0.667	-0.010	-0.441	1.098
**Ni@1T-MoS_2**	0.495	-0.011	-0.441	0.925
**NiO1@1T-MoS_2**	-0.106	-0.011	-0.441	0.324
**NiO2@1T-MoS_2**	0.107	-0.010	-0.441	0.538
	**Δ*E*(H*)/eV**	**Δ*ZPE*(H*)/eV**	**Δ*TS*(H*)/eV**	**Δ*G*(H*)/eV**
**1T-MoS_2**	0.458	0.078	-0.162	0.698
**Fe@1T-MoS_2**	0.046	0.082	-0.163	0.291
**Co@1T-MoS_2**	0.004	0.081	-0.162	0.247
**Ni@1T-MoS_2**	-0.081	0.078	-0.161	0.158
**NiO1@1T-MoS_2**	-0.784	0.078	-0.162	-0.544
**NiO2@1T-MoS_2**	-0.427	0.079	-0.162	-0.186

Supplementary Table 7 Comparison of HER performance in 1.0 M KOH solution for NiO@1T-MoS_2/CFP with other MoS_2-based HER electrocatalysts

Catalyst	Tafel slope (mV dec^{-1})	η_{10}[a] (mV)	Loading mass (mg cm^{-2})	References
**Ni@1T-MoS_2/CFP**	**52**	**46**	**1.02**	**This work**
Ni-MoS_2	60	98	0.89	9
MoS_2/Ni_3S_2	83	110	9.7	10
Ni-Co-MoS_2	51	155	0.286	11
Ni-Co/1T-MoS_2	38	70	0.15	12
MoSP/CB[b]	120	57	0.28	13
HF-MoSP	85	119	0.35	14
T-MoS_2	78	290	0.05	15
2HMoS_2-Si/SiO_2	50	100[c]	-	16
MoS_2/N-RGO-180	41.3	56	0.14	17
SV-MoS_2	60	170	-	18
MoS_2 film[b]	70	~200	-	19
MoS_2@N-doped C	55	165	1	20
$NiMo_3S_4$	98	257	0.3	21
M-MoS_2	41	175	0.043	22
MoS_2/MoO_2	76.1	240	0.22	23
MoS_2/WS_2[b]	69	~ 250	0.035	24
Co-doped MoS_2[b]	110	~230	-	25
Co-Mo-S_x[d]	-	~205	0.05	26
Ni-Mo-S/C[e]	85.3	200	0.52	27
MoS_2-S180[b]	55	200	0.285	28
MoS_2/$CoSe_2$[b]	36	68	0.28	29

[a] represents the overpotential (η) at the current density of 10 mA cm^{-2}; [b] represents HER performance in 0.5 M H_2SO_4 solution. [c] represents the onset potential (η). [d] the overpotential (η) at the current density of 5 mA cm^{-2} in 0.1 M KOH. [e] the overpotential (η) at the current density of 10 mA cm^{-2} in pH ~ 7. all potentials versus RHE.

Supplementary Methods

The calculations of TON and TOF

The hexagonal NiO@1T-MoS_2 units in NiO@1T-MoS_2/CFP nanosheet is assumed to be the catalytic active sites. According to the elemental analysis result, the molar mass of NiO@1T-MoS_2 is ~ 1294 g mol^{-1}. Thus, the loading mass of 1.02 mg cm^{-2} for Ni@MoS_2/CFP is equal to a loading mole of 0.79 μmol·cm^{-2} for NiO@1TMoS_2 units on CFP. Chronoamperometry (CA) shows a stable hydrogen evolution current-time plot over 30 h in 1.0 M KOH (~107 mA cm^{-2} at an overpotential of -130 mV, inset of Fig. 5d). Assuming the Faradaic efficiency is 100% and the electrode area is 1 cm^2, a turnover number (TON) of 75600 and turnover frequency (TOF) of 0.70 s^{-1} could be obtained after 30 h. See the following equation (1) for calculation details:

$$\mathrm{TOF} = \frac{107\,\frac{mA}{cm^2} \times 1cm^2 \times \frac{1A}{1000mA} \times \frac{1\,^{C}/_{s}}{1A} \times \frac{1mol}{96485\,C} \times \frac{1mol\,H_2}{2mol}}{7.9 \times 10^{-7}\,mol\,Catalysts} = 0.70\,s^{-1}$$

$$\mathrm{TON} = \mathrm{TOF} \times 60 \times 60 \times 30\,s = 75600 \qquad \text{........(1)}$$

DFT calculations

Density functional calculations are performed within the generalized gradient approximation (GGA) and Perdew-Burke-Ernzerhof (PBE) exchange-correlation functional[30] as implemented in VASP code[31-33]. The electron-ion interaction is described with projector-augmented plane wave (PAW) potentials[34,35]. For all calculations, the plane-wave energy cutoff is set to 520 eV and the energy convergence threshold is set as 10^{-5} eV. Monkhorst-Pack k-point meshes (with spacing $2\pi \times 0.03$ Å^{-1}) are employed for the full relaxation of geometry structures. The atomic flake and its neighboring image are separated by a vacuum space that exceeds 15 Å. The van der Waals (vdW) interactions are based on the optB86b-vdW function. Calculations on HER free energy diagram were performed at the surface of monolayer structures. The actual reaction mechanism in alkaline HER follows the following key steps:

$$\mathrm{cat} + H_2O + e^- \rightarrow H^*@cat + OH^-\ (Volmer\ step) \qquad (1)$$

$$2H^*@cat \rightarrow H_2\uparrow\ (Tafel\ step) \qquad (2)$$

$$H^*@cat + H_2O + e^- \rightarrow cat + OH^- + H_2\uparrow\ (Heyrovsky\ step) \qquad (3)$$

The free energies for step (1) and step (3) should be the same at HER equilibrium potential. Under this assumption, computations on the exact free energy of OH^- in solutions could be avoided by using computational hydrogen elextrode[36]. The free energies for each step are calculated as equation (4).

$$\Delta G = \Delta E + \Delta ZPE - T\Delta S \tag{4}$$

Where, ΔG, ΔE, ΔZPE, $T\Delta S$ are the changes for free energy, enthalpy from DFT calculations, zero-point energy and entropy (T = 300 K), respectively. ΔZPE may be derived after frequency calculation by equation (5)[37]:

$$ZPE = \frac{1}{2}\sum h v_i \tag{5}$$

Where h is the Planck constant, v_i are the computed vibrational frequencies.

The TS values of adsorbed species are calculated with the vibrational frequencies, as shown in equation (6)[38]:

$$TS_{v_i} = k_B T\left[\sum_i \ln\left(\frac{1}{1 - e^{-hv_i/k_bT}}\right) + \sum_i \frac{hv_i}{k_B T}\frac{1}{(e^{hv_i/k_bT})} + 1\right] \tag{6}$$

Where k_B is the Boltzmann constant, T is the temperature. TS values of H_2 and H_2O are from previous reports[39].

Supplementary References

1. Nomiya, K. *et al.* Anderson-type heteropolyanions of molybdenum(VI) and tungsten(VI). *Polyhedron* **6**, 213-218 (1987).
2. Yu, H. *et al.* Transition-Metal-Controlled Inorganic Ligand-Supported Non-Precious Metal Catalysts for the Aerobic Oxidation of Amines to Imines. *Chem. Eur. J.* **23**, 13883-13887 (2017).
3. Ravel, B. Newville, M. ATHENA, ARTEMIS, HEPHAESTUS: data analysis for X-ray absorption spectroscopy using IFEFFIT. *J. Synchrotron. Radiat.* **12**, 537-541 (2005).
4. Dickinson, R.G. and Pauling, L. The Structure of Molybdenite. *J. Am. Chem. Soc.* **45**, 1466-1471 (1923).
5. Fang, Y. *et al.* Structure Re-determination and Superconductivity Observation of Bulk 1T MoS2. *Angew. Chem. Int. Ed.* **57**, 1232-1235 (2018).
6. Rooksby, H.P. Structure of Nickel Oxide. *Nature* **152**, 304 (1943).
7. Fang, H. et al. Degenerate n-Doping of Few-Layer Transition Metal Dichalcogenides by Potassium. *Nano Lett.* **13**, 1991-1995 (2013).
8. Xu, E.Z. *et al.* p-Type transition-metal doping of large-area MoS2 thin films grown by chemical vapor deposition. *Nanoscale* **9**, 3576-3584 (2017).
9. Zhang, J. *et al.* Engineering water dissociation sites in MoS2 nanosheets for accelerated electrocatalytic hydrogen production. *Energy Environ. Sci.* **9**, 2789-2793 (2016).
10. Zhang, J. *et al.* Interface Engineering of MoS2/Ni3S2 Heterostructures for Highly Enhanced Electrochemical Overall-Water-Splitting Activity. *Angew. Chem. Int. Ed.* **55**, 6702-6707 (2016).
11. Yu, X.Y. *et al.* Formation of Ni-Co-MoS2 Nanoboxes with Enhanced Electrocatalytic Activity for Hydrogen Evolution. *Adv. Mater.* **28**, 9006-9011 (2016).

12. Li, H. *et al.* Amorphous nickel-cobalt complexes hybridized with 1T-phase molybdenum disulfide via hydrazine-induced phase transformation for water splitting. *Nat. Commun.* **8**, 15377 (2017).
13. Ye, R. *et al.* High-Performance Hydrogen Evolution from MoS2(1-x) P(x) Solid Solution. *Adv. Mater.* **28**, 1427-1432 (2016).
14. Wu, A. *et al.* Hierarchical MoS2@MoP core-shell heterojunction electrocatalysts for efficient hydrogen evolution reaction over a broad pH range. *Nanoscale* **8**, 11052-11059 (2016).
15. Liu, Y. *et al.* Self-optimizing, highly surface-active layered metal dichalcogenide catalysts for hydrogen evolution. *Nature Energy* **2**, 17127 (2017).
16. Voiry, D. *et al.* The role of electronic coupling between substrate and 2D MoS2 nanosheets in electrocatalytic production of hydrogen. *Nat. Mater.* **15**, 1003-1009 (2016).
17. Tang, Y.-J. *et al.* Molybdenum Disulfide/Nitrogen-Doped Reduced Graphene Oxide Nanocomposite with Enlarged Interlayer Spacing for Electrocatalytic Hydrogen Evolution. *Adv. Energy Mater.* **6**, 1600116 (2016).
18. Li, H. *et al.* Activating and optimizing MoS2 basal planes for hydrogen evolution through the formation of strained sulphur vacancies. *Nat. Mater.* **15**, 364 (2016).
19. Li, G. *et al.* All The Catalytic Active Sites of MoS2 for Hydrogen Evolution. *J. Am. Chem. Soc.* **138**, 16632-16638 (2016).
20. Yu, X.Y. *et al.* Ultrathin MoS2 Nanosheets Supported on N‐doped Carbon Nanoboxes with Enhanced Lithium Storage and Electrocatalytic Properties. *Angew. Chem. Int. Ed.* **54**, 7395-7398 (2015).
21. Jiang, J. *et al.* Hollow Chevrel-Phase NiMo3S4for Hydrogen Evolution in Alkaline Electrolytes. *Angew. Chem. Int. Ed.* **55**, 15240-15245 (2016).
22. Geng, X. *et al.* Pure and stable metallic phase molybdenum disulfide nanosheets for hydrogen evolution reaction. *Nat. Commun.* **7**, 10672 (2016).
23. Yang, L. *et al.* Porous metallic MoO2-supported MoS2 nanosheets for enhanced electrocatalytic activity in the hydrogen evolution reaction. *Nanoscale* **7**, 5203-5208 (2015).

24. Xu, S.; Li, D. Wu, P. One-Pot, Facile, and Versatile Synthesis of Monolayer MoS2/WS2 Quantum Dots as Bioimaging Probes and Efficient Electrocatalysts for Hydrogen Evolution Reaction. *Adv. Funct. Mater.* **25**, 1127-1136 (2015).
25. Wang, H. *et al.* Transition-metal doped edge sites in vertically aligned MoS2 catalysts for enhanced hydrogen evolution. *Nano Res.* **8**, 566-575 (2015).
26. Staszak-Jirkovský, J. *et al.* Design of active and stable Co–Mo–Sx chalcogels as pH-universal catalysts for the hydrogen evolution reaction. *Nat. Mater.* **15**, 197-203 (2015).
27. Miao, J. *et al.* Hierarchical Ni-Mo-S nanosheets on carbon fiber cloth: A flexible electrode for efficient hydrogen generation in neutral electrolyte. *Sci. Adv.* **1**, e1500259 (2015).
28. Xie, J. *et al.* Controllable Disorder Engineering in Oxygen-Incorporated MoS2 Ultrathin Nanosheets for Efficient Hydrogen Evolution. *J. Am. Chem. Soc.* **135**, 17881-17888 (2013).
29. Gao, M.R. *et al.* An efficient molybdenum disulfide/cobalt diselenide hybrid catalyst for electrochemical hydrogen generation. *Nat. Commun.* **6**, 5982 (2015).
30. Perdew, J.P.; Burke, K.; Ernzerhof, M. Generalized Gradient Approximation Made Simple. *Phys. Rev. Lett.* **77**, 3865-3868 (1996).
31. Kresse, G.; Furthmüller, J. Efficiency of Ab-Initio Total Energy Calculations for Metals and Semiconductors Using a Plane-Wave Basis Set. *Comput. Mater. Sci.* **6**, 15-50 (1996).
32. Kresse, G.; Furthmüller, J. Efficient Iterative Schemes for Ab Initio Total-Energy Calculations Using a Plane-Wave Basis Set. *Phys. Rev. B* **54**, 11169-11186 (1996).
33. Kresse, G.; Hafner, J. Ab Initiomolecular Dynamics for Liquid Metals. *Phys. Rev. B* **47**, 558-561 (1993).

34. Kresse, G.; Hafner, J. Ab Initiomolecular-Dynamics Simulation of the Liquid-Metal–amorphous-Semiconductor Transition in Germanium. *Phys. Rev. B* **49**, 14251-14269 (1994).

35. Kresse, G.; Joubert, D. From Ultrasoft Pseudopotentials to the Projector Augmented-Wave Method. *Phys. Rev. B* **59**, 1758-1775 (1999).

36. Blöchl, P. E. Projector Augmented-Wave Method. *Phys. Rev. B* **50**, 17953 (1994).

37. Zheng, Y. *et al.* High Electrocatalytic Hydrogen Evolution Activity of an Anomalous Ruthenium Catalyst. *J. Am. Chem. Soc.* **138**, 16174-16181 (2016).

38. Nørskov, J. K. *et al.* Trends in the Exchange Current for Hydrogen Evolution. *J. Electrochem. Soc.* **152**, J23-J26 (2005).

39. Bendavid, L. I.; Carter, E. A. CO2 Adsorption on Cu2O(111): A DFT+U and DFT-D Study. *J. Phys. Chem. C* **117**, 26048-26059 (2013).
